# Debates in allergy medicine: Molecular allergy diagnosis with ISAC will replace screenings by skin prick test in the future

**DOI:** 10.1186/s40413-017-0162-3

**Published:** 2017-09-19

**Authors:** E. Jensen-Jarolim, A. N. Jensen, G. W. Canonica

**Affiliations:** 1 Institute of Pathophysiology and Allergy Research, Center of Pathophysiology, Infectiology and Immunology, Medical University of Vienna, Vienna, Austria; 2 The interuniversity Messerli Research Institute, University of Veterinary Medicine Vienna, Medical University Vienna, University of Vienna, Vienna, Austria; 3 AllergyCare, Allergy Diagnosis and Study Center, Vienna, Austria; 4 Personalized Medicine Asthma & Allergy Clinic, Humanitas University & Research Hospital, Rozzano, Milan, Italy

## Abstract

In today’s clinical practice patients’ skin is used as screening organ for diagnosing type 1 allergy. According to European guidelines skin prick testing with a panel of 18 allergen extracts is recommended, in the US between 10 to 50 allergens are used. The specificity and sensitivity of skin testing is individually highly variable depending on age, body mass, and skin barrier status. In atopic inflammation skin testing gives more false positive results. Smaller skin area and strain limits prick testing in small children. Although the risk for systemic reactions in skin prick testing is very small, emergency medications must be available. Considering the fact that IgE is the only reliable biomarker for type I allergy, upfront IgE screening with ISAC, followed by fewer skin tests to approve positive sensitizations, is proposed. It is time to arrive in the age of molecular allergy diagnosis in daily patient care.

## Background

Since its detection, specific IgE represents the only diagnostic biomarker for exposure and sensitization in allergy [1] with predictive value in asthma [2], and of value for selecting patients for allergen immunotherapy [3]. It reliably correlates with clinical symptoms in respiratory allergies, less in food allergies, and is usually interpreted in the context of skin prick tests [4]. In most cases during daily clinical practice, IgE-determinations as well as skin prick tests are done with allergen extracts. In both cases, results must be interpreted considering the clinical symptoms and history of the patient, as even inhalant sensitization not necessarily correlates with symptoms [5]. Allergen extracts are produced under good laboratory practice (GLP) conditions by incubation of allergen sources (pollen from pollen farms, cultured house dust mites, food) in aqueous buffer solutions, followed by filtering and purification steps. As a result, the extracts contain a variety of allergens (e.g. Bet v 1 a), besides non-allergenic proteins or isoallergens non-relevant for IgE binding (e.g. Bet v 1d, e). Production severely depends on the allergen sources and associated environmental, culture and ripening conditions, which makes standardization of allergen extracts a difficult task. To improve the quality of extracts, it is accepted that they may sometimes be “spiked” with singe allergen molecules [6]. Variations in the biological activity of allergen extracts are decisive for in vitro IgE testing and skin prick tests, and even more when extracts are applied as therapeutics for allergen immunotherapy [7]. Comparisons to reference extracts have been requested for a long time [8]. In Europe, allergens for diagnostic application fall under the directives for therapeutics [9]. The cost-intensive procedures of approval and maintenance of approved products led to a dramatic reduction of diagnostic allergens available for intradermal use [10], but also for skin prick allergens a diagnostic bottle neck is to be expected. The improved standards of diagnostic and therapeutic allergens may critically affect allergy diagnosis in the near future and prompts the critical evaluation of the fidelity and reliability of alternative methods.

## Skin prick testing – allergy screening in the skin

Skin prick tests are regarded as means to determine sensitization and should be interpreted in the light of clinical history, clinical picture and results of testing for specific IgE. According to the American College of Allergy, Asthma and Immunology (ACAAI) [11], 10**–**50 allergen extracts are used for skin prick testing. The European guidelines propose a panel of 18 respiratory allergens of which, simultaneously, improved standardization is encouraged [12]. While skin prick testing in respiratory allergies is a reliable diagnostic tool, in food allergy more false positive results are seen on the one hand, while on the other hand over 95% of patients negative in skin prick tests with food do not present with immediate type symptoms [13]. The skin prick results should be compared to the positive control prick with histamine dihydrochloride 10 mg/ml [14]. The calculation of a histamine equivalent prick -index (HEP) area may be helpful, where the allergen prick size is correlated to the size of the histamine wheal to define a cut off value, but it is proposed that the true area of the wheal “is theoretically more accurate” than the diameter [15]. Like the Immuno solid-phase allergen chip (ISAC) -test, therefore, also the skin prick test (SPT) is a “semiquantitative” method. The wheal size of allergen skin prick tests has been associated with the extent of clinical reactivity especially in adults [16], and was suggested a predictive marker for clinical reactivity to specific food allergens, for instance for albumin at a diameter of 9 mm, for yolk 7, for cow’s milk or fresh cow’s milk 20 or 1 mm, respectively [17].

It should be noted that the histamine prick result itself is individually variable and depends on age and body mass index of the patient [18]. This finding was approved in a Korean study where obese children had significantly larger histamine wheals [19]. In contrast, the histamine tests in atopic children resulted in a significantly smaller flare, but longer itch reaction [20].

Importantly, the mean wheal diameter resulting from the prick has been shown to be affected by the personnel testing and by the lancet weight [21], and is differing between test centers, naturally depending on the concentration of the histamine solution used: a 1 mg/ml solution with wheals between 3 and 6.8 mm was found unacceptable, the form and size of lancets used resulted in comparable analytical sensitivities and specificities, and pain scores [22].

The collected data thus document continuous efforts to improve the fidelity of skin prick tests which individually vary depending not only on the patient, but even more on the assistants doing the test and on the exactness of the recording method.

## There are disadvantages in skin prick testing

Anaphylactic side effects are a concern when testing with biologically active allergens in vivo and the possibility of emergency treatment must be provided [23]. In the largest cohort so far investigated with this specific question, 31,000 patients, in 0.077% systemic side reactions were recorded, with the highest risk with peanut and nuts when the wheal diameter was of over 8 mm [24]. The risk for systemic reactions due to skin tests treated by epinephrin i.m. evaluated in 1456 patients was totally 3.6% (intradermal testing: 3.1%; skin prick testing 0.41%), and highest in females [25]. A study in 20,530 patients reported that 80 patients tested experienced systemic reactions, 13 of them more severe, and calculated a risk of 0.009 and 0.003% for experiencing a major reaction during skin prick testing [26].

It is accepted that several conditions may elevate the risk for systemic reactions in skin prick testing, like previous anaphylactic events, testing in small children and in pregnancy with a risk for mother and child, and in uncontrolled asthma [27]. It is also known that the higher number of skin tests in polysensitized patients needed for diagnosis is associated with a higher risk for adverse reactions [28]. Larger skin prick test reactions and associated enhanced risk for adverse reactions have been explained by enhanced permeability of the skin [28].

## Conditions reducing skin prick test reliability

It has been reported that stress in the patient may sporadically lead to false positive skin reactivity [29], but more studies are needed to support these observations. However, what is much more important in clinics is that the intake of numerous medications may interfere with skin prick reactivity. This was recently analysed in detail in a large retrospective study [30]. Tricyclic antidepressants, benzodiazepines, quetiapine, and mirtazapine should be discontinued 1 week before testing, H(1)-blockers 3 days [30]. The risk for a negative histamine test was not elevated for selective serotonin reuptake inhibitors, selective norepinephrine reuptake inhibitors and proton pump inhibitors [30]. Therefore, the abstinence from drugs needs to be planned in advance of skin prick testing, but it is often a difficult task to take patients off their medications even over a few days. IgE testing is not dependent on any interferences with medications.

## Why doctors and patients like skin prick testing

Albeit the many practical limitations as reviewed above, it seems impossible to abstain from skin prick tests in daily management of allergic patients. In most incidences skin prick tests are done upfront further allergy diagnosis as they allow a readout within 15**–**20 min. Physicians of any specialty apply skin prick tests, especially as costs are refunded by health insurances and they do not require expensive devices but only well-trained personnel. They know well that skin prick tests visually and dramatically document to the patients the existing hypersensitivity. This is very useful for patient compliance concerning further allergy tests and therapy. A patient who has undergone repeated skin tests is no longer fond of this method and tends to reject repeated testing.

Skin prick testing in an epidemiological study achieved a 90% compliance in school children when re-testing after 10 years [31] and allowed an estimation of prevalence of sensitization rising from 30 to 41%. Positive skin prick tests in newborns predicted an allergic career to early adult age [32]. However, when skin test with house dust mite allergens was evaluated in 692 patients, it was found most reliable only in patients below 50 years of age [33]. This is problematic as allergies occur in elderly to a similar rate as in younger adults, and must be diagnosed, as reviewed previously [34].

The interpretation of skin prick test results on atopic skin, which may be false positive, actually requires an expert in order to prevent unnecessary avoidance diets [35]. When Foong et al**.** compared head-to-head skin prick testing and IgE testing in atopic children, there was no difference in food-specific results, but in respiratory allergies specific IgE testing resulted in more (false) positive results than prick testing or ISAC IgE testing [36]. Also before, ISAC testing in atopic children has been found to be a promising alternative overcoming problems of testing in the hypersensitive atopic skin, but still correlating well with the skin tests [37].

## Molecular allergy diagnosis goes global

In contrast to natural allergen extracts and purified allergens, recombinant allergens can be expressed under standardized conditions without undesired contamination, with an exactness matching the today’s requirements of diagnostic allergens.

Overall, the accumulating knowledge on molecular allergens has changed our understanding of allergic mechanisms and helped to design sensitization maps all over the world [38], and even establish correlations with climate change [39]. Equally important, molecular allergy, particularly multiplex allergen microarray diagnosis proved successful globally, such as in Spain [40], Italy [41], in the overall Mediterranean area [42], Iran [43], South Africa [44, 45], Brazil [46], and in China [47].

Molecular allergy diagnosis using singleplex allergens or multiplex allergen microarrays are typical methods of precision medicine [48] and they enhance the specificity of IgE-diagnosis in polysensitized respiratory allergies [49], can be applied in food allergies [36, 50] and atopic eczema [36, 37], and may even reveal unexplained anaphylaxis [3]. A strong correlation was found between results with the ISAC112 microarray test, and SPT and other specific IgE tests [51, 52], with a particularly good correlation in allergies to pollen [53] and to house dust mites [54]. It is accepted that molecular allergy diagnosis improves the risk evaluation, sorts out genuine from cross-reactive sensitizations, improves the overall predictive value of the diagnostic results, as well as the accuracy of the resulting allergen immunotherapy. In daily routine maximally 112 allergens can be tested at a time, but in experimental approaches more than 170 molecules have proven possible [55]. Technically much more will be possible in the future, considering that impressively half of the published 3000 allergens in the Allergome data base (www.allergome.org) are available in natural or recombinant form.

Considering the rapid development of molecular allergy during the past 3 decades, and relating it to the complexity of nature, we may only asymptotically approach harboring “all” allergens for diagnosis. This is even more true for therapeutic allergens. In terms of clinical diagnosis, this limitation may for the time being be circumvented by prick-to-prick testing with suspected (and suspicious) substances brought by the patient.

## Recommendations and praxis: molecular allergy entered clinics

As a shift in paradigm, the WAO-ARIA-GA2LEN consensus document [56], which is presently updated, states that molecular-based allergy diagnostics, may be used by the expert in the second-line diagnostic workup, thus equivalent with extract-based skin prick- and IgE-testing. It has to be emphasized that any allergy diagnostic method, including IgE and SPT screening, may render unexpected results, which have to be handled in the light of the patient’s history and clinical picture. For the less experienced allergists, automated tools were developed to support the complex interpretations of over 100 results [57], whereas the classical method due to the subjective bias in the doctor’s investigation renders a simplified, but possibly incomplete view. Hence, the diagnostic allergy field is in transition at the moment, and a first “Molecular Allergology User’s Guide” was urgently needed as recently published by the European Academy of Allergy and Clinical Immunology (EAACI) [58]. In this handbook, besides the classical diagnostic work-up “from symptoms to molecules” (Top-down) starting off with extract-based skin prick screening and IgE-testing, the procedure “from molecules to clinic” (bottom-up) is discussed, which starts with allergen molecule-related information followed by the other tests. Considering that most doctors in allergy diagnosis will not leave the skin prick method as a primary screening approach, the authors proposed the “U-shaped” approach as a compromise, integrating both methods [58].

One major argument against the bottom-up approach is usually the economic constraint.

## Are the economic concerns against ISAC relevant?

At present few clinics routinely apply component-resolved diagnosis using allergen microarrays. In most cases this method is offered to the patient as a private service when all other diagnostic workup has been completed. This is on the one hand due to economic restrictions as most health insurances do not cover the costs of the ISAC allergen microarray testing. Therefore, the ISAC test is offered to more affluent patients. This economic perspective is the likely reason for the gender bias towards more male patients visiting the a private allergy center offering ISAC as first line diagnostics. It is well known that the socioeconomic and health insurance status affects the access to medical care also in totally other fields of medicine [59]. A recent meta-analysis predicted that microarray testing could be cost-saving only if a substantial reduction of single IgE testing and oral food challenge tests could be achieved. Simultaneously, the authors could not identify microarray studies resulting in changes in patient management significant enough to render cost-reductions [60].

Cost disadvantages of ISAC may have to do with i) multiplex IgE testing taking more time to interpret and communicate the results to the patient, but also by ii) the general habit of using the microarray as the final allergy diagnosis method, instead of using it for screening. This results in an enhancement of the cumulative costs.

Especially in polysensitized patients the ISAC allergen microarray could lead to a cost reduction [58]. In contrast to the procedure “from symptoms to molecules”, starting at the skin as primary screening organ followed by 2-step IgE screening, the “from molecules to clinic” approach is more timely and therefore economically interesting for patients, doctors and health insurances.

## Conclusion

From the above research it becomes apparent that skin prick testing is a historic compromise and has many disadvantages, such as impreciseness, operator- and patient- dependency, and the risk for systemic reactions, albeit in the % to ‰ range. Nobody has so far dared to address any potential de novo sensitization through skin prick testing. This is remarkable since it has been known for a long time [61], and new evidence is accumulating that the skin is a highly effective route for sensitization, even more so in settings of barrier disruption, sometimes even rendering anaphylaxis [62].

Furthermore, we conclude that allergy screening with the ISAC multiplex allergen array not only with a similar fidelity leads to allergy diagnosis, but is favorable in

- polysensitized patients
- in small children with limited skin area, but higher strain
- in elderly when skin tests get less reliable [34].
- In all settings of inflamed or atopic skin
- when medications interfering with skin prick testing cannot be discontinued

ISAC testing has a high sensitivity and specificity [38], and showed a strong correlation with singleplex tests including IgE and skin prick testing with extracts [51, 52], specifically for respiratory allergens [53, 54], with slight alterations from allergen to allergen.

We strongly believe that in the future, skin prick screening will no longer be acceptable for allergy diagnosis, considering the more stringent recent regulations. Allergy diagnosis should finally arrive in the twenty-first century and start with ISAC as one of the most comprehensive methods and using IgE as the unique biomarker for allergies. It is clear that – in analogy to the classical procedure starting with skin prick test screening, results must under any circumstances be aligned with the clinical picture. However, upfront IgE screening followed by fewer selected SPTs in relation to the clinical phenotype, will reduce the strain in the tested patient, whilst still following the international standards.

## Abbreviations

ACAAI: American College of Allergy, Asthma and Immunology
Ig: Immunoglobulin
ISAC: Immuno solid-phase allergen chip
SPT: Skin prick test
